# Parenteral Formulation of Zopiclone

**DOI:** 10.4103/0250-474X.40342

**Published:** 2008

**Authors:** P. V. Swamy, P. Sushma, G. Chirag, K. Prasad, M. Younus Ali, S. A. Raju

**Affiliations:** Department of Pharmaceutics, HKES' College of Pharmacy, Sedam Road, Gulbarga - 585105, India

**Keywords:** Zopiclone, solubility enhancement, parenteral formulation, shelf-life, accelerated stability

## Abstract

The present study was undertaken with an intention to develop a stable and effective parenteral formulation, containing the drug zopiclone. Since zopiclone is a water insoluble drug, various methods such as co-solvency, pH control and hydrotrophy have been tried in order to enhance its solubility. When all these methods could not give adequate solubility enhancement of the drug, a hydrochloride salt was prepared, and it was found to be thermostable. Various batches of zopiclone hydrochloride injection formulation were prepared in order to assess the influence of light, atmospheric oxygen and antioxidant on the stability of the drug and the formulations were also subjected to accelerated stability testing in order to predict approximate shelf-life of the product.

In spite of a few limitations such as pain on injection and inconvenient administration requiring a trained person, parenteral formulations are much widely used especially when an immediate physiologic response is needed in life-threatening emergencies and for administering those drugs that are destroyed by digestive secretions. On the other hand, parenteral drug delivery systems are useful for administering drugs to non-cooperative, nauseous or unconscious patients.

Zopiclone (ZP) is a non-benzodiazepine sedative hypnotic used for the short term treatment or management of insomnia^1^. It is also having anticonvulsant, antiaggressive and anticonflict actions in addition to its sedative and hypnotic effects. The onset of action with oral dosage forms is relatively slow. Therefore in order to facilitate its administration to non-cooperative aggressive psychotic patients to induce sleep and for arresting seizures in epileptic patients, the present study was undertaken to develop a stable and effective parenteral formulation containing ZP.

ZP was procured from Kumud Drugs Pvt Ltd, Sangli, India. Polyethylene glycol 400, propylene glycol, ethylenediamine tetraacetic acid, glycerin, hydrochloric acid, sodium meta bisulphite and urea were procured from S. D. Fine Chem., Boisar, India. Methanol and ethanol were procured from Ranbaxy Chemicals, New Delhi, India and E-Merck (India) Ltd, Mumbai, respectively. All the other chemicals were of analytical reagent grade.

ZP is a poorly water soluble drug. To make a clear and thermodynamically stable solution, solubility studies were performed in distilled water along with other cosolvents, such as polyethylene glycol (PEG-400), propylene glycol (PG), glycerin, 10% v/v ethanol adjusted to various pH conditions, viz., 4.5, 4.0 and 3.5 (Table 1). Excess amount of ZP was added to the stoppered 100 ml conical flasks containing 10 ml of solvent and was shaken for 6 h on a gyratory flask shaker (100 rpm). Then they were removed from the shaker and kept aside for 24 h at room temperature to attain equilibrium. Suitable aliquots were withdrawn from the filtered solutions and analyzed for drug content spectrophotometrically at 303.6 nm against appropriate solvent blank^2^. The experiments were run in triplicate.

**TABLE 1:** SOLUBILITY STUDIES

Solvent	Saturation solubility (μg/ml)
Distilled water	115
5% v/v PEG 400	195
10% v/v PEG 400	475
0.1N HCl	1650
5% v/v Propylene glycol	350
20% v/v Propylene glycol	580
5% v/v Glycerine	300
10% v/v Glycerine	340
20% v/v Glycerine	450
5% v/v Ethanol	280
10% v/v Ethanol	440
20% v/v Ethanol	440
10% v/v Ethanol (pH 4.5)	515
10% v/v Ethanol (pH 4.0)	860
10% v/v Ethanol (pH 3.5)	1120

Since adequate solubility (5 mg/ml) of the drug could not be achieved for the formulation of an injection, the hydrochloride salt^3^,^4^ was prepared by dissolving 2 g of ZP in about 100 ml of methanol and 2.5 ml of dilute hydrochloric acid (2.3 ml HCl made to 10 ml with distilled water) was added. The solution was kept on water bath and evaporated to dryness. The residual zopiclone hydrochloride (ZPHCl) was collected and stored in a desiccator.

For the formulation of injection, ZPHCl (1.1 g) was dissolved in sufficient water for injection (WFI) in order to get 200 ml solution. The pH of the solution was found to be 4.5. The above solution (2.15 ml) was filled in 2 ml ampoules, sealed and immediately sterilized by autoclaving (30 min at 121°). For estimation of drug content, the injection formulation (0.5 ml) was appropriately diluted with distilled water and the absorbance was measured at 303.6 nm using distilled water as blank on a UV/Vis spectrophotometer (Shimadzu-1700). Average of three determinations was taken as the drug content of the formulation. The drug obeys Beer Lambert's law in the concentration range of 4-20 μg/ml^5^.

The effect of oxygen, light and temperature on the formulation was studied by storing the ampoules under various conditions for a period of 5 w. To test the effect of oxygen, the injection (2.15 ml) was filled in each 2 ml and 10 ml capacity ampoules. The air in 10 ml capacity ampoule was not displaced before sealing (condition ‘A’), whereas the air present in the 2 ml capacity ampoule was replaced by flushing with carbondioxide and sealed (condition ‘B’). Samples from both sets of ampoules were withdrawn periodically at 5 d intervals and the drug content was estimated. Two sets of ampoules were used to study the effect of light on the formulation (Batch-I, Table 2. The first set of ampoules was wrapped in aluminum foil and kept in a dark place. The second set of ampoules was stored in such a manner that they were exposed to daylight. The drug content of the samples was estimated periodically at intervals of 5 d. To determine the effect of temperature on the formulation, sufficient number of ampoules filled with the formulation were stored at different temperatures i.e., 4° (refrigerator), 30° (room temperature), 55° and 75°. Samples were withdrawn at intervals of 5 d, and the content of drug remaining was estimated spectrophotometrically.

**TABLE 2:** CODING OF VARIOUS BATCHES OF ZOPICLONE INJECTION

Code name	Air replacement	Antioxidant
Batch-I	-	-
Batch-II	Yes	-
Batch-III	Yes	0.01% w/v disodium EDTA
Batch-IV	Yes	0.1% w/v ascorbic acid

For determination of sleeping time^6^, Swiss mice (*n* = 9) weighing between 25-30 g were selected and numbered accordingly. They were divided into three groups. Group-I served as control. ZPHCl was injected intraperitoneally to group-II and group-III and the time of administration was noted. The animals were observed for the onset and duration of action. Since the animals did not show onset of action at the calculated dose of 13 μg and at double the calculated dose, a dose of 50 μg was administered. Dose for mice was calculated using the formula, human dose × mice factor, i.e. 5000 × 0.0026 = 13 μg.

ZP is practically insoluble in water; various techniques such as cosolvency, pH control and hydrotrophy have been attempted. Sufficient solubility of the drug (5 mg/ml) could not be achieved by these methods (Table 1). Since ZP is a weak base, a hydrochloride salt with the required solubility has been prepared and was used for the formulation of injection. The prepared salt form of ZP i.e., ZPHCl was found to withstand autoclaving (percent drug lost during autoclaving is nil). Therefore, autoclaving was selected as the method of choice for sterilization. Different batches of ZPHCl injection formulation were prepared in order to assess the influence of various parameters such as light, atmospheric oxygen and antioxidants on the stability of the drug. Four of the formulations (Batch-I to Batch -IV, Table 2) were subjected to accelerated stability testing by storing the samples at 4° (refrigerator), 30° (room temperature), 55° and 75° over a period of 40 d and the data obtained was presented in Tables 3 and 4. Results of the present study indicated that oxygen and light have appreciable effect on the stability of ZP parenteral formulations. The percent drug lost was less in ampoules stored at 4° and 30° (maximum 19.53% at 30° in 40 d) whereas, it was very high in the ampoules stored at higher temperatures i.e., 55° and 75° (upto 70.17% at 75° in 40 d). Disodium EDTA and ascorbic acid as antioxidants (along with air replacement) did not enhance the stability of the formulation. On the other hand, the former has deleterious effect on its stability. In the presence of ascorbic acid, the formulation developed a purple colour, which may be due to the formation of coloured complex (chromophor).

**TABLE 3:** KINETIC DATA OF VARIOUS BATCHES OF ZOPICLONE INJECTION FORMULATIONS

Temperature	k(day^−1^)			
	
	Batch-I	Batch-II	Batch-III	Batch-IV
04°	0.0017	0.0010	0.0017	0.0013
30°	0.00432	0.0026	0.0041	0.0028
55°	0.0172	0.1680	0.0207	0.0120
70°	0.0506	0.0368	0.0299	0.0370

k is first-order degradation constant; batch-I is zopiclone injection formulation without air replacement and antioxidant; batch-II is with air replacement and without antioxidant; batch-III is with air replacement and 0.01% w/v disodium EDTA; batch-IV is with air replacement and 0.1% w/v ascorbic acid

**TABLE 4:** DATA FROM ARRHENIUS PLOTS

Batch	Correlation coefficient (r)	k_25_	Shelf-life (days)	

			4°	25°
I	−0.9997	0.00303	61.3	34.7
II	−0.9905	0.00196	108.8	53.7
III	−0.9676	0.00351	62.9	29.9
IV	−0.9998	0.00193	84.0	54.4

k is first-order degradation constant at 25°; batch-I is zopiclone injection formulation without air replacement and antioxidant; batch-II is with air replacement and without antioxidant; batch-III is with air replacement and 0.01% w/v disodium EDTA; batch-IV is with air replacement and 0.1% w/v ascorbic acid

Intraperitoneal injection of the formulation in albino mice (*n* = 9) shows faster onset of action and longer duration of sleeping time compared to oral route of administration. The injection shows 16 and 60 min values respectively, for above parameters against the 28 and 40 min values of oral route.

Replacement of air above the solution in the container (ampoule) with an inert gas gives a shelf-life of about three and a half months when stored in a refrigerator. The present study concludes that ZPHCl gives adequate solubility for the drug, thus making it possible to develop a parenteral formulation in an aqueous medium.
